# Digital Whole Slide Image Analysis of Elevated Stromal Content and Extracellular Matrix Protein Expression Predicts Adverse Prognosis in Triple-Negative Breast Cancer

**DOI:** 10.3390/ijms25179445

**Published:** 2024-08-30

**Authors:** Zsófia Karancsi, Barbara Gregus, Tibor Krenács, Gábor Cserni, Ágnes Nagy, Klementina Fruzsina Szőcs-Trinfa, Janina Kulka, Anna Mária Tőkés

**Affiliations:** 1 Department of Pathology Forensic and Insurance Medicine, Semmelweis University, 1091 Budapest, Hungary; gregus.barabara@stud.semmelweis.hu (B.G.);; 2 Department of Pathology and Experimental Cancer Research, Semmelweis University, 1085 Budapest, Hungarynagy.agnes@semmelweis.hu (Á.N.); 3 Department of Pathology, Albert Szent-Györgyi Faculty of Medicine, University of Szeged, 6725 Szeged, Hungary; 4 Department of Pathology, Bács-Kiskun County Teaching Hospital, 6000 Kecskemét, Hungary

**Keywords:** triple-negative breast cancer, tumor microenvironment, tumor–stroma ratio, prognostic factors, digital image analysis

## Abstract

Triple-negative breast cancer (TNBC) is a subtype of breast cancer with a poor prognosis and limited treatment options. This study evaluates the prognostic value of stromal markers in TNBC, focusing on the tumor–stroma ratio (TSR) and overall stroma ratio (OSR) in whole slide images (WSI), as well as the expression of type-I collagen, type-III collagen, and fibrillin-1 on tissue microarrays (TMAs), using both visual assessment and digital image analysis (DIA). A total of 101 female TNBC patients, primarily treated with surgery between 2005 and 2016, were included. We found that high visual OSR correlates with worse overall survival (OS), advanced pN categories, lower stromal tumor-infiltrating lymphocyte count (sTIL), lower mitotic index, and patient age (*p* < 0.05). TSR showed significant connections to the pN category and mitotic index (*p* < 0.01). High expression levels of type-I collagen (>45%), type-III collagen (>30%), and fibrillin-1 (>20%) were linked to significantly worse OS (*p* = 0.004, *p* = 0.013, and *p* = 0.005, respectively) and progression-free survival (PFS) (*p* = 0.028, *p* = 0.025, and *p* = 0.002, respectively), validated at the mRNA level. Our results highlight the importance of stromal characteristics in promoting tumor progression and metastasis and that targeting extracellular matrix (ECM) components may offer novel therapeutic strategies. Furthermore, DIA can be more accurate and objective in evaluating TSR, OSR, and immunodetected stromal markers than traditional visual examination.

## 1. Introduction

Invasive breast cancer is a complex and heterogeneous disease and is one of the leading causes of cancer-related deaths worldwide [1,2]. Based on the expression of hormone receptors (estrogen and progesterone receptors), human epidermal growth factor receptor 2 (HER2), and the Ki-67 proliferation marker, breast carcinomas are further categorized into hormone-receptor (HR) positive (the Luminal A- and B-like), HER2-positive, and triple-negative breast cancer (TNBC) subtypes [3]. This study focuses on the particularly aggressive TNBCs, a subset with limited biomarkers and treatment options due to the lack of targeted therapies.

While the clinical impact of several aspects associated with tumor cells is well-known, less is understood about how the tumor microenvironment (TME) affects tumor behavior. Thus, there is a need to better understand the stromal characteristics and structure to elucidate the connection between tumor cells and different stromal components, which could enable the development of new treatments or prevention strategies. Subtype-specific remodeling of the tumor–stroma could explain the differences in cellular malignancy and response to therapy among different breast cancer subtypes [4].

Emerging evidence shows that the tumor–stroma ratio (TSR), defined as the percentage of stroma relative to the neoplastic cells in tumor tissue, has independent prognostic value in several solid tumors. Initially described in colon cancer [5], high TSR scores were also later confirmed in breast cancer [6], nasopharyngeal cancer [7], esophageal squamous cell carcinoma [8], early cervical carcinoma [9], epithelial ovarian cancer [10], and hepatocellular carcinoma [11], etc., as a negative prognostic marker [12]. The adverse prognostic role of high TSR scores has also been demonstrated, and its standard determination both in surgical and core biopsy samples elaborated in breast cancer, including TNBC [13,14,15,16]. TSR is usually determined on hematoxylin and eosin (H&E)-stained sections through visual examination or machine learning-based digital image analysis (DIA) involving artificial intelligence (AI)-based approaches [17,18,19,20]. We presumed that reliable detection of extracellular matrix components could facilitate the rapid and accurate testing of TSR values using DIA.

The TSR method focuses exclusively on the most desmoplastic area of the representative surgical specimen slide. The majority of studies have used tissue microarrays (TMA) to examine the extent of the stroma in selected areas of the tumor tissue [19,21,22]. However, the stromal content of the entire tumor area has been scarcely examined in the literature. One article focusing on TNBC indicated that a high stromal content determined on whole slide images (WSI) with AI was associated with worse survival [23].

The TME consists of various non-malignant cells, an altered extracellular matrix (ECM), and soluble factors such as growth factors and cytokines [24,25]. Among the several components of the ECM, collagens and fibrillins are of particular interest due to their structural and functional roles in both normal tissue and pathological conditions [26,27].

Type-I collagen is the most abundant type of collagen in the human body and is a major structural component of the ECM. Type-I collagen has a dual role in cancer survival. It acts as a barrier to early tumor invasion and can restrict cancer cell growth, as evidenced by studies where the deletion of the *COL1A1* gene led to increased metastasis formation. Conversely, type-I collagen can promote tumor growth by providing structural support to cancer cells and a physical barrier to leukocytes. Type-I collagen furthermore contributes to the epithelial–mesenchymal transition and upregulates the expression of matrix metalloproteinases [28,29]. High type-I collagen level generally correlates with worse outcomes in cancers, including breast cancer [30,31,32,33].

Type-III collagen often coexists with type-I collagen and is involved in the early stages of wound healing and tissue repair [34]. Its presence in the TME is most likely coupled to tissue remodeling and fibrosis, which is common in aggressive tumors [35,36,37]. It has been shown that silencing the *COL1A1* gene leads to favorable tumor characteristics in TNBC [38]. Additionally, the upregulation of the *COL1A1* and *COL1A1* genes, among others, has been observed in breast cancers with brain metastases. Similarly to type-I collagen, findings about type-III collagen’s effect on prognosis are disparate, with some studies linking high type-III collagen protein expression with worse prognosis [32], and others suggest a tumor-suppressive role [35].

Fibrillin-1 is a glycoprotein that forms microfibrils and is critical for the structural integrity and elasticity of connective tissues. In cancer, fibrillin-1 regulates TGF-β signaling, a key pathway in tumor progression and metastasis [26,39,40]. However, its effect on tumor progression in the case of breast cancer is to be further analyzed.

In this study, we aimed to evaluate the potential prognostic value of stromal ECM biomarkers in TNBC utilizing both visual assessment and DIA. We especially focused on the evaluation of TSR and overall stroma ratio (OSR) based on WSIs and type-I collagen, type-III collagen, and fibrillin-1 expression detected on TNBC TMAs. Our goal was to set up and validate a reproducible method that can be smoothly integrated into the routine pathology workflow to provide relevant prognostic information. Also, the potential association of these proteins with clinicopathological parameters and outcomes in TNBC may offer valuable insight to be traced further on their roles in the molecular mechanisms of tumor development and metastasis.

## 2. Results

### 2.1. Clinicopathological Characteristics of Patients

Our cohort consisted of 101 female patients diagnosed with TNBC, primarily treated with surgery between 2005 and 2016. The median age was 59 years (range 33–91). During the study period, 38/101 (38%) patients were deceased. Disease progression occurred in 38/101 (38%) patients; of these, 12/101 (12%) had locoregional recurrence, and 31/101 (31%) developed distant metastasis. In patients with both locoregional and distant relapse, the timing of the initial progression event was documented. The median overall survival (OS) was 92 months (range 7–220), and the median progression-free survival (PFS) was 90 months (range 5–220).

Most cases were histological grade 3 (99/101) and of no-special type invasive breast cancer (76/101). The median stromal tumor-infiltrating lymphocytes (sTIL) ratio was 20% (range 0–90%), and the median mitotic index was 26 (range 6–105). According to the 8th edition of UICC TNM staging [41], the distribution of tumor size and nodal status were as follows: 4 pT1b; 50 pT1c; 41 pT2; 2 pT3; 49 pN0; 6 pN1mi; 25 pN1a; 4 pN2a; 1 pN3a (Table 1, Figure S1). Among patients, 74/101 (74%) received adjuvant chemotherapy.

### 2.2. Variability and Categorization of TSR and OSR on H&E-Stained WSIs

Out of the 101 patients, WSIs were available for 99 cases. The intra- and interobserver variability of the determination of TSR (Table 2A) and OSR (Table 2B) scores were good or excellent and significant in both the visual and DIA-assessed scores.

Using the 50% cut-off point recommended by Mesker et al. [5], 65% of cases were categorized as stroma high (SH) based on visual TSR evaluation, and this increased to 67% with DIA. Utilizing the same cut-off point for OSR, 22% of cases were SH visually, compared to 30% with DIA.

We used the median scores as cut-off points for further analysis and calculations to distribute our cases evenly. The cut-off points were 65% for visual TSR, 65.05% for DIA TSR, 35% for visual OSR, and 39.34% for DIA OSR.

Additionally, we noted that 17% of the tumors contained fibrotic foci, and 46% exhibited necrosis extending beyond a single field of ×100 magnification.

### 2.3. Association of the TSR and the OSR with the Clinicopathological Factors

OSR was strongly associated with age (t = 3.5, *p* < 0.001); patients younger than 48 years (17/101) all had a stromal percentage below 40%. A similar tendency was observed for TSR (t = 1.8, *p* = 0.07). Neither TSR nor OSR was associated with the pT category. However, there was a significant correlation between TSR and pN category when comparing pN0-pN1mic to pN1a-pN3a (t = 3.2, *p* = 0.002). Similar results were found for OSR (t = 2.5, *p* = 0.013). Overall, higher stromal content was more frequently associated with macrometastatic nodal involvement (Table 1, Figure S1).

A low OSR was associated with higher sTIL values (t = 3.0, *p* = 0.004). We found a significant negative correlation between the mitotic index (MI) and both the OSR (t = 3.7, *p* < 0.001) and the TSR (r = −0.28, *p* = 0.009) when dividing MI into two groups at the value of 25 according to optimal binning (Table 1, Figure S1).

Tumors with fibrotic foci had significantly higher OSR (t = 3.5, *p* < 0.001) and TSR values (t = 3.9, *p* < 0.001). Tumors with large necrotic areas were associated with lower OSR (t = 3.3, *p* = 0.001) and TSR (t = 2.2, *p* = 0.033) values.

### 2.4. Impact of TSR and OSR on Survival

**Figure 1:**
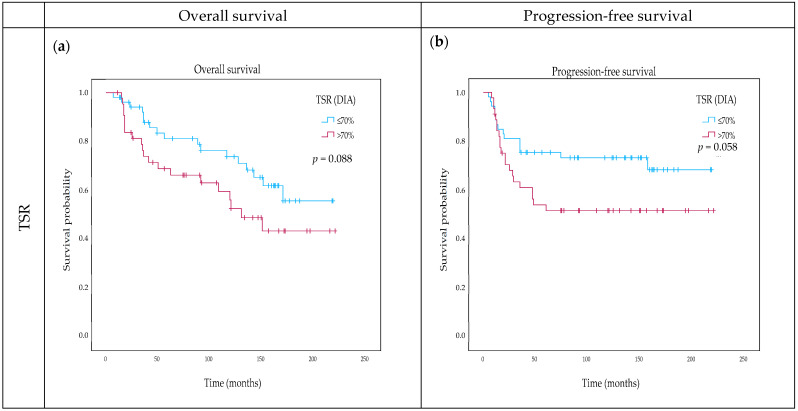

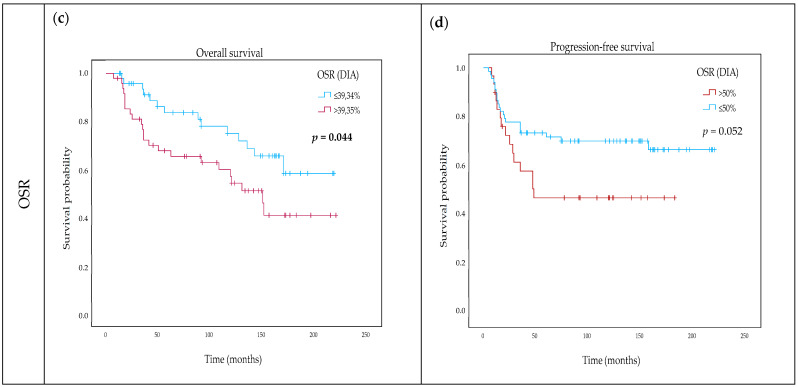
Overall stromal ratio (OSR) and tumor–stroma ratio (TSR) in relation to overall survival (OS) (**a**,**c**) and progression-free survival (PFS) (**b**,**d**) utilizing best cut-off determined with multiple Log-Rank analysis. *p*-values below 0.05 are in bold.

OS showed a significant correlation with DIA OSR, clustered according to the median at 39.34% (*p* = 0.044) using Kaplan–Meier analysis. Visual OSR also showed a significant correlation at the median cut-off of 35% (*p* = 0.017). High stroma content was linked to worse survival (Figure 1c). TSR was not significantly associated with OS, with DIA TSR showing a stronger correlation, indicating a trend toward worse survival above 70% scores (*p* = 0.088) (Figure 1a). Additional characteristics that we found to be associated with worse OS included the presence of necrosis (*p* = 0.032), treatment without adjuvant chemotherapy (*p* = 0.048), and sTIL below 20% (*p* = 0.036).

When applying the univariate Cox regression model, we found a significant connection between the OS and OSR (HR = 1.023, *p* = 0.023), age, number of positive lymph nodes, presence of necrosis, and clustered sTIL. In the multivariate analysis, the following variables were included: DIA OSR, age, mitotic index, number of positive lymph nodes, and sTIL. High DIA OSR (HR = 1.037, *p* = 0.028) and high mitotic index (HR = 1.040, *p* = 0.002) were associated with worse OS in multivariate analysis. The remaining variables, such as TSR, did not predict OS with significance in the Cox regression model (Table 3).

When analyzing PFS, higher OSR tended to be associated with worse survival at the 50% cut-off, utilizing the Kaplan–Meier curve (*p* = 0.052) (Figure 1d). TSR was only associated with PFS at a 70% cut-off point (*p* = 0.058) (Figure 1b). Further factors that negatively influenced the PFS were the presence of lymph node metastasis (*p* = 0.001), fibrotic foci (*p* = 0.042), and sTIL value below 20% (*p* = 0.04).

Utilizing univariate Cox regression analysis, we found a significant connection between the PFS and the number of positive lymph nodes, the presence of fibrotic foci, and the clustered sTIL. High DIA OSR tended to be associated with worse PFS (HR = 1.907, *p* = 0.058). The following variables were included in the multivariate analysis: DIA OSR, number of positive lymph nodes, and the presence of fibrotic foci. The high number of positive lymph nodes was significant for worse PFS (HR = 1.179, *p* = 0.002). We did not find a substantial connection between PFS and TSR or OSR in univariate or multivariate Cox regression analysis (Table 3).

### 2.5. Variability and Categorization of Type-I Collagen, Type-III Collagen, and Fibrillin-1 Expression and Stromal Content on TMAs

**Figure 2:**
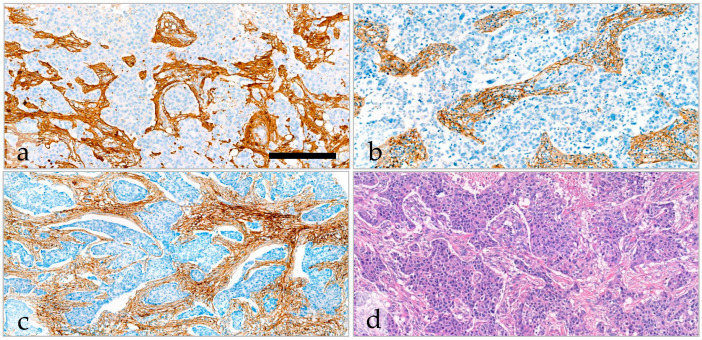
Immunostaining for extracellular matrix components, type-l collagen (**a**), type-III collagen (**b**), and fibrillin-1 (**c**) accurately highlights the tumor–stroma seen on the H&E stained section (**d**) of TNBC. The scale bar represents 200 µm on (**a**,**b**,**d**) and 300 µm on (**c**).

Sensitive automated immunostaining of ECM proteins allowed the precise detection of the tumor–stroma intermingled with the tumor tissue (Figure 2; see Section 4). The correlation between the percentage of positivity determined by DIA PatternQuant (PQ) and visual examination was excellent and significant in the case of every immunohistochemical staining (ICC > 0.927, *p* < 0.01) (Table S1). The correlation between the visually determined intensity scores and the clustered DIA DensitoQuant (DQ) scores was fair or slight (ICC_type-III collagen_ = 0.566, ICC_fibrillin-1_ = 0.398, *p* < 0.01) (Table S2). We used the PQ and DQ scores determined with DIA for further calculations. The median values and the cut-off points of each immunoreaction used for survival analyses are described in detail in Table 4. The correlation between the expression of these ECM proteins and the clinicopathological characteristics is shown in Figure S1.

There was a significant, good correlation between the PQ values of the different immunoreactions. Furthermore, the correlation between the type-I collagen and H&E-based results (stromal ratio) was also good and significant (r = 0.864, *p* < 0.001). The results encountered from the TMAs resembled the OSR moderately (Table 5).

### 2.6. The Effect of Type-I Collagen, Type-III Collagen, and Fibrillin-1 Expression on Survival

**Figure 3:**
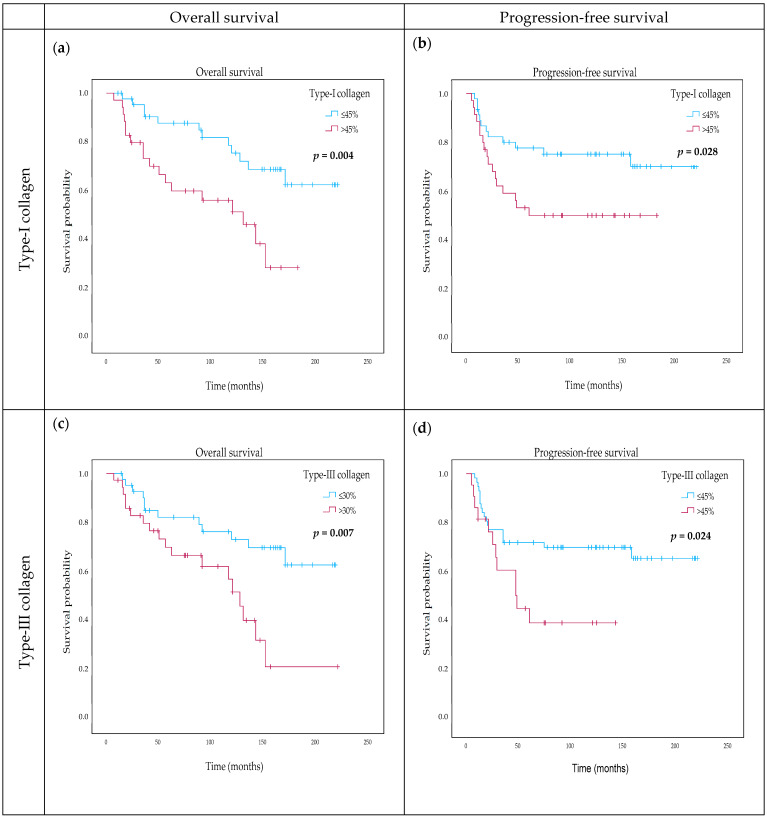

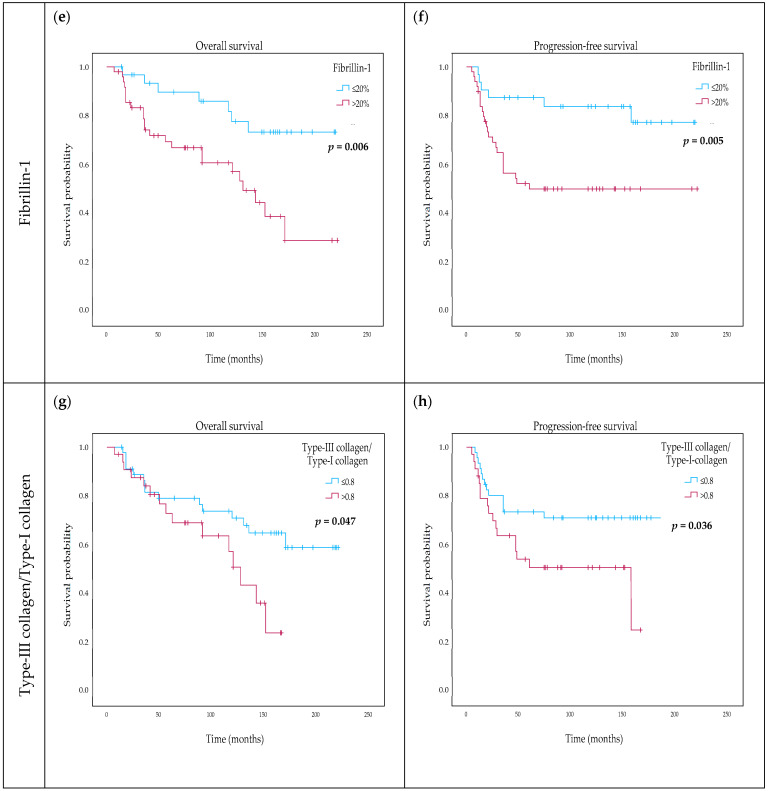

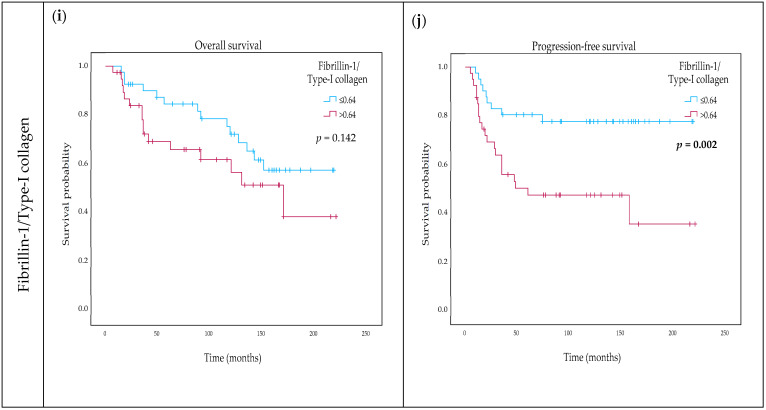
Overall survival and progression-free survival in relation to type-I collagen (**a**,**b**), type-III collagen (**c**,**d**), and fibrillin-1 (**e**,**f**) positivity and to the type-III collagen/type-I collagen (**g**,**h**) and fibrillin-1/type-I collagen (**i**,**j**) ratio utilizing best cut-off determined with multiple Log-Rank analysis. *p*-values below 0.05 are in bold.

Type-I collagen was significantly associated with OS, using the best cut-off at 45% (Table 5): 26% (12/46) of the patients with ≤45% type-I collagen extent died compared to the patients with >45% type-I collagen extent, with 51% (18/35) deaths (*p* = 0.004) (Figure 3a). Furthermore, with the same cut-off point, high type-I collagen expression was significantly associated with a higher progression probability of 49% (17/35) compared to the 26% (12/46) (*p* = 0.028) of the low type-I collagen group (Figure 3b).

Type-III collagen showed similar results as type-I collagen. OS was worse among patients with type-III collagen-rich (>30%) tumors (49%, 18/37) compared to the type-III collagen-poor (≤30%) tumors (28%, 12/43) (*p* = 0.007) (Figure 3c). In the case of PFS, we found a significant correlation with progression using a higher cut-off point of 45%; there were 12/22 (55%) progressions among the tumors with high type-III collagen expression compared to the low-expressing tumors where 31% (18/58) progressed (Figure 3d). The intensity scores of the immunoreactions did not correlate with survival, whether assessed visually or using DIA. However, the multiplied value of DQ and PQ showed a significant correlation with OS (*p* = 0.013) and PFS (*p* = 0.025) using the cut-off point of 7600.

High fibrillin-1 expression was also associated with significantly worse survival. Fourty-six % (23/50) deaths were recorded in the fibrillin-1-high group compared to the fibrillin-1-low group, where 22% (7/32) of the patients died, dividing the scores at 20% (*p* = 0.006) (Figure 3e). Furthermore, PFS was also strongly associated with fibrillin-1 using the 20% cut-off point: 24/50 (48%) progression was reported in the fibrillin-1-high and 6/32 (19%) in the fibrillin-1-low group (*p* = 0.005) (Figure 3f). Neither intensity scores nor DQ*PQ values showed a correlation with clinical outcomes.

To account for the possibility that our high fibrillin-1 and type-III collagen scores merely resulted from a higher stroma content, we analyzed the survival according to the type-III collagen/type-I collagen and fibrillin-1/type-I collagen ratios. We found that a type-III collagen/type-I collagen ratio above 0.81 is linked with worse OS (*p* = 0.047) and PFS (*p* = 0.036) (Figure 3g,h). Additionally, a fibrillin-1/type-I collagen ratio above 0.64 is significantly associated with shorter PFS (*p* = 0.002) (Figure 3i,j). Fibrillin-1/type-III collagen was not associated with survival.

Lastly, we identified cases with high expression levels of both type-I collagen (>50%) and type-III collagen (>30%) and compared these to cases with lower expression levels of each protein. We found a significant correlation with OS (*p* < 0.001) and PFS (*p* = 0.036). The average OS time was notably different, with 90 months in the high-expression group compared to 173 months in the low-expression group. Similarly, the average PFS time was 85 months in the high-expression group versus 164 months in the low-expression group.

We conducted univariate Cox regression analysis and found that type-I collagen, type-III collagen, and fibrillin-1 are indicators of poor prognosis. We validated these results the way we included each of these proteins separately in multivariate analyses along with the same clinicopathological parameters as age, mitotic index, number of positive lymph nodes, and sTIL the univariate Cox regression results for these parameters are presented in Table 3. For clarity, we have only presented the results of the separate multivariate tests for the stromal proteins in Table 6.

**Figure 4:**
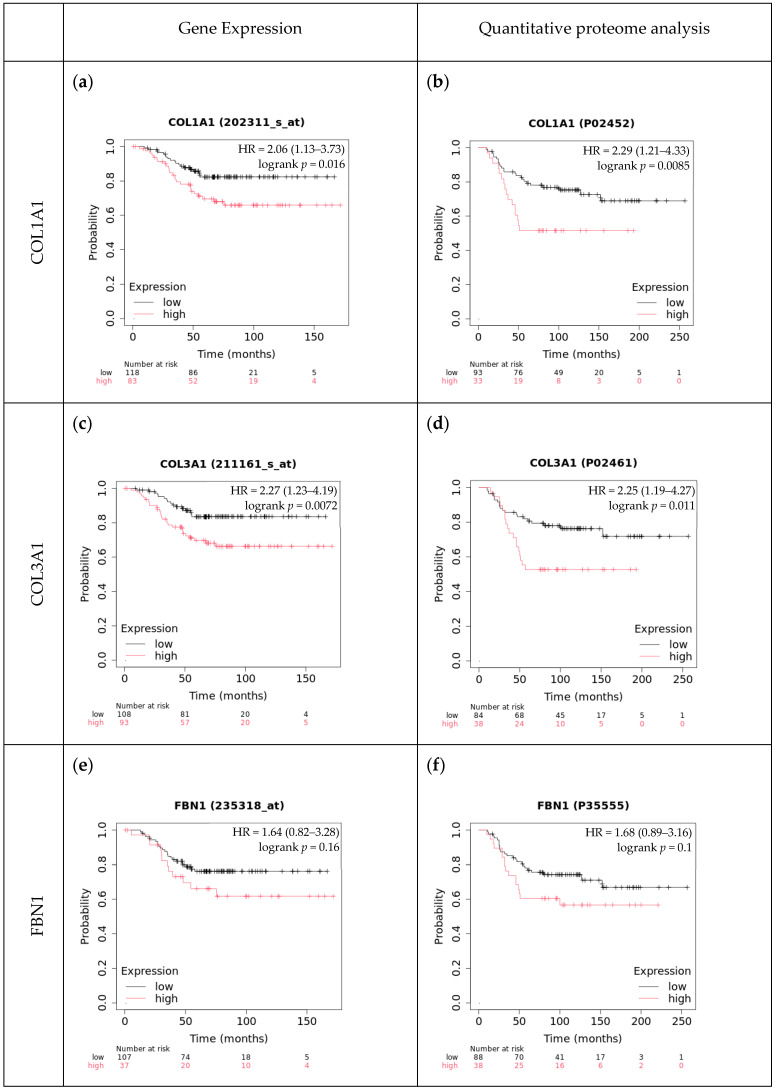
Overall survival in relation to type-I collagen (**a**,**b**), type-III collagen (**c**,**d**), and fibrillin-1 (**e**,**f**) mRNA and protein expression: Kaplan–Meier Plotter analysis (https://kmplot.com/analysis/ (accessed on 10 June 2024)).

To validate our findings, we used the Kaplan–Meier Plotter database, an online survival analysis tool, and found mostly similar results [42]. For type-I collagen and type-III collagen, high expression levels of both mRNA and the protein were associated with significantly worse OS probability. However, when analyzing fibrillin-1 in these cohorts, we found no significant correlation between gene or protein expression and OS (Figure 4). Considering RFS (recurrence-free survival) data, high mRNA expression levels of fibrillin-1 were associated with a significantly worse prognosis. Similarly, high protein expression levels of both type-I collagen and type-III collagen were linked to significantly lower RFS probability.

## 3. Discussion

### 3.1. Discussion of Study Results

TNBC of no special type is the most aggressive molecular subtype of breast cancer, with few biomarkers available for risk stratification or therapy planning. In recent decades, the tumor–stroma has gained attention in oncological research because phenotypic and genotypic classifications of cancer cells alone cannot fully explain tumor behavior and clinical outcomes. The TME consists of various non-malignant cells, an altered ECM, and soluble factors such as growth factors and cytokines. While normal breast stroma is the key to breast differentiation, cancer-associated stromal cells can create a permissive microenvironment for tumorigenesis, enhancing angiogenesis, tumor cell proliferation, survival, invasion, and metastasis [24]. Although changes in the tumor–stroma are recognized as crucial factors in tumor progression, this aspect has yet to be fully incorporated into routine clinical decision-making.

The TSR was developed as a quantitative method to measure the ratio of all resident TME components compared to the tumor cells. The distribution of TSR values in TNBC varies according to the 50% cut-off point determined by Mesker et al. [5]. Some studies report that 60% of cases are stroma-high (SH), while others report 40% [15,16]. According to our results, 65% of TNBC cases were categorized as SH. Studies agree with the negative prognostic effect of high TSR in TNBC [43]. Here, we did not find significant results, although TSR above 70% indicated a tendency toward worse OS (*p* = 0.088) and PFS (*p* = 0.058).

The TSR method focuses exclusively on the most desmoplastic area of the tumor’s representative slide, potentially overlooking the significance of the entire tumor’s stromal content. Few studies on colorectal carcinoma have linked high stromal content on WSI, with a threshold of 50%, to worse survival outcomes [44,45]. Additionally, a study using AI on TNBC WSIs found that patients with tumors characterized by high stromal content had worse survival outcomes [23]. Similarly, we found that a higher OSR is associated with worse OS at a median score of 39.34% (*p* = 0.044) and PFS at 50% (*p* = 0.052). This result suggests that unselective analysis of the WSI can hold important information regarding the prognostic value of the stroma.

Studies analyzing stromal content on TMAs consistently show that high stromal content is associated with a worse prognosis [19,22]. One study analyzed TMAs stained with cytokeratin and found that the optimal cut-off value for the negative prognostic effect of stroma percentage was 33.5% [22]. Another article presented that a stroma ratio of 50% and above is significantly associated with a worse prognosis [19]. However, Micke, P. et al., analyzing the stroma content of 16 solid tumor types, including 50 cases of TNBC, found that stroma-low tumors had a worse prognosis [21].

Breast cancer progression is characterized by a gradual increase in collagen deposition, linearization, and thickening of collagen fibers, all of which result in increased ECM stiffness [46]. Several collagen types, including collagen type-I, -II, -III, -V, and -IX, exhibit increased deposition as the disease advances [32]. Closer analysis of the stroma in breast cancer subtypes has revealed that HER2-positive and TNBC exhibit significantly more heterogenous and stiffer ECM compared to the luminal subtypes [46]. Considering that these subtypes present the highest mortality risk for breast cancer patients and are linked to a notably higher relapse rate, it is crucial to understand how the stroma and tissue mechanics influence this aggressive behavior [47].

Our results on immunostained TMAs suggest promising new biomarkers in TNBC. Type-I collagen appears to be a universal stromal marker and is expected to serve as a negative prognostic factor. In various cancers, including breast cancer, type-I collagen has been identified as a cancer-promoting factor, influencing cell proliferation, metastasis, apoptosis, cisplatin resistance, and overall cancer progression and prognosis [48]. While most studies confirm its negative impact on prognosis [31,32,33], a few have reported it as a positive prognostic indicator [49]. In our study, the abundance of type-I collagen showed a strong negative effect on OS (*p* = 0.004) and PFS (*p* = 0.028) at a cut-off of 45% positivity. Accordingly, it has been previously suggested that type-I collagen could become a potential therapeutic target, as its degradation may attenuate the stiffness of the matrix and simultaneously contribute to more efficient drug delivery into solid tumors [50].

Similarly, type-III collagen has been linked to both favorable [35] and poor prognosis [32]. We found a clear connection between elevated collagen-III levels (with a 30% threshold) and shortened OS (*p* = 0.013) and (with a 45% threshold) with shortened PFS (*p* = 0.025). To account for the possibility that high stromal content caused the elevated type-III collagen levels and their associated negative prognostic effect, we calculated the type-III collagen/type-I collagen ratio. We found that values above 0.8 were associated with worse OS (*p* = 0.047) and PFS (*p* = 0.036).

The effect of fibrillin-1 expression in the TME of breast cancer on prognosis is less understood. However, the fibrillin-1 gene (FBN1) has been found to be upregulated in various cancers, including gastric cancer, osteosarcoma, and ovarian cancer [51]. The online TNMplot gene expression analysis presented significant upregulation of the FBN1 gene when comparing normal breast tissue to breast tumor gene chip data [52]. Our results indicate that high fibrillin-1 expression (i.e., above 20%) is strongly linked to worse PFS (*p* = 0.002) and OS (*p* = 0.005) divided at 20%. Similarly to type-III collagen, we calculated the fibrillin-1/type-I collagen ratio and found a strong association with worse PFS above 0.64 (*p* = 0.002) but not with OS (*p* = 0.142).

OSR was significantly associated with age, pN category, sTIL, and mitotic index (*p* < 0.05). TSR showed significant connections to the pN category and mitotic index (*p* < 0.01). In multivariate survival analysis, OSR demonstrated a significant independent effect on OS (HR = 1.955, *p* = 0.045), while TSR did not correlate with OS or PFS. Both type-I collagen (HR = 3.075, *p* = 0.01 for OS; HR = 2.725, *p* = 0.017 for PFS), type-III collagen (HR = 3.467, *p* = 0.007 for OS; HR = 3.826, *p* = 0.011 for PFS), and fibrillin-1 (HR = 3.439, *p* = 0.017 for OS; HR = 3.815, *p* = 0.018 for PFS) significantly influenced both OS and PFS.

Our results, obtained through DIA, demonstrate that QuantCenter, a commercial module integrated with 3DHISTECH’s SlideViewer software, is a valuable tool for objectively analyzing digitized slides. The strong correlation between visual and DIA-assisted scores on H&E-stained slides highlights its effectiveness in differentiating stroma and tumor compartments. Most DIA tools measuring stroma extent in breast cancer are capable of distinguishing the same three clusters we used: tumor, stroma, and fat/background [19,21]. However, this approach has limitations, such as difficulty in differentiating necrosis from tumor cytoplasm and lymphocytes from tumor nuclei due to color similarities, necessitating manual exclusion of these elements. AI-assisted tools could provide a solution as they have shown promising results, identifying up to nine clusters on histology slides [23,44,45].

DIA proved effective for analyzing immunostained slides, as the distinct colors allowed easy identification of different clusters. This accuracy was corroborated by the strong correlation observed between the visual assessments and the DIA-assisted scores, as well as between the DIA scores from both observers. Because of this, for the punctual and objective evaluation of the TME, we recommend the utilization of DIA on slides stained with a universal stromal marker such as type-I collagen. Other research examining breast TMAs used cytokeratin to mark the tumor areas [22]. Detailed pixel-by-pixel analysis of the tissue can help with the objective measurement of staining intensity, which is quite subjective even between trained pathologists with visual examination [53].

### 3.2. Limitations of This Study

The retrospective nature of our research resulted in incomplete data for certain clinicopathological characteristics, such as pN category and mitotic index. The relatively small sample size may affect the generalizability of our findings. A further limitation that should be mentioned is that only one slide per tumor was analyzed, which may not fully account for tumor heterogeneity, potentially leading to variable results with different slides. Additionally, there is a lack of sufficient prior research on topics concerning fibrillin-1 and overall stromal content determined on whole slide images, which limits the context and comparison of our results. The technical limitations associated with DIA have already been discussed in the first part of the Discussion. Further studies with larger cohorts and more comprehensive data are needed to validate our findings and expand the knowledge in this area.

## 4. Materials and Methods

### 4.1. Patients

One hundred and one TNBC cases treated between 2005 and 2016 at the Bács-Kiskun County Teaching Hospital in Kecskemét were included in the study. None of the patients received neoadjuvant chemotherapy prior to surgery. Cases were pT1–pT3 and M0 tumors, and sufficient follow-up data was available.

Patients’ records were collected from the electronic medical database of the Bács-Kiskun County Teaching Hospital in Kecskemét in accordance with the following Ethical Approval: ETT TUKEB: BMEÜ/443-5/2022/EKU. The collected clinicopathological characteristics included age, patient follow-up information, survival data (OS, PFS), histological type, mitotic index, pT, tumor size, pN, number of metastatic lymph nodes, sTIL, and whether the patient received adjuvant chemotherapy.

### 4.2. Study Design

Four-micrometer-thick sections of 99 TNBC cases, routinely H&E-stained slides representing the whole tumor, were used to evaluate the TSR and OSR.

**Figure 5:**
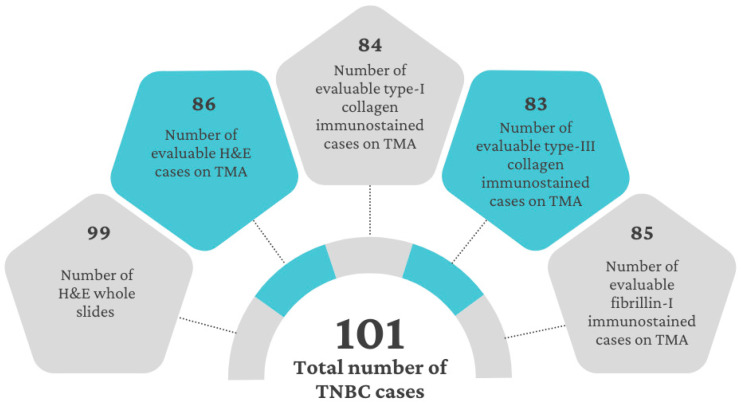
Number of evaluable cases considering stromal content and immunostainings.

**Figure 6:**
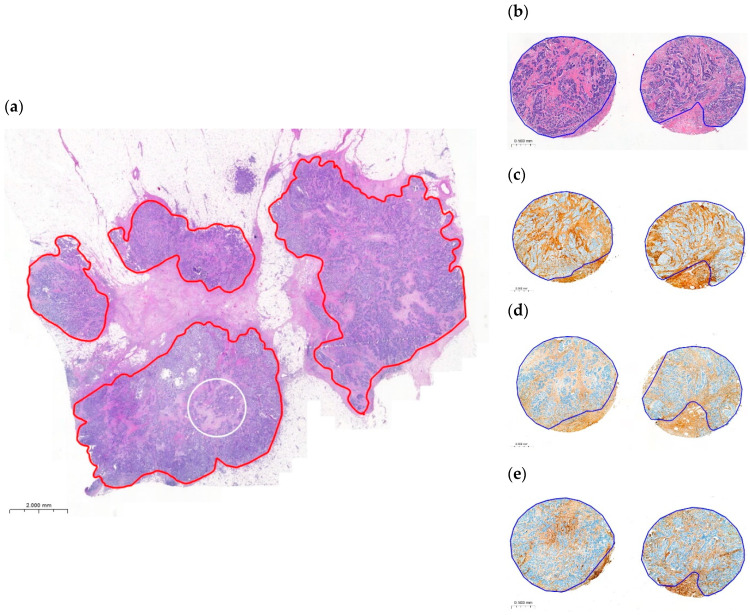
Annotations used for the analysis of the same tumor are illustrated. On the whole tumor slide, the red annotation marks the area where the OSR was determined, while the white annotation indicates a 2 mm diameter circle representing the area where the TSR was assessed (**a**). Additionally, we utilized H&E (**b**), type-I collagen (**c**), type-III collagen (**d**), and fibrillin-1 (**e**) stained TMAs of the same tumor.

For further evaluation, TMAs were constructed. Subsequently, 2–4 cores, each 2 mm in diameter, were sampled from each tumor block. For immunohistochemical analysis, 3 μm thick serial sections were cut from each TMA block. IHC reactions were performed with a Ventana Benchmark Ultra automated instrument (Roche Diagnostics, Tucson, AZ, USA), including antigen retrieval in the high pH CC1 buffer for 40 min, incubation with the primary antibodies for 60 min, detection with the Ultraview system for 40 min and visualization with a DAB/hydrogen peroxide kit. The following primary antibodies were used: rabbit polyclonal anti-human type-I collagen (1:1000, #PA5-95137, ThermoFisher/Invitrogen, Waltham, MA, USA); anti-mouse/human type-III collagen (1:100, #600-401-105-01, ThermoFisher/Rockland, Rockford, IL, USA); and mouse monoclonal anti-human fibrillin 1 (1:800, immunoglobulin G1 (IgG1), clone:26, MAB2502, Merck/Chemicon, Darmstadt, Germany) IgGs. After hematoxylin counterstaining, dehydration, and clearing, the sections were mounted using a Pertex medium [54]. Additionally, one slide from each of the four TMAs was stained with H&E. The number of evaluable cases varied across the different stainings; 86 H&E stained, 84 type-I collagen, 83 type-III collagen, and 85 fibrillin-1 immunostained cases were available (Figure 5 and Figure 6).

After the staining process, slides were scanned using a Pannoramic 1000 scanner (3DHISTECH Ltd., Budapest, Hungary). Visual analysis and digital scoring were performed using the SlideViewer program v2.5 (3DHISTECH) with the Quantcenter v2.3 module.

### 4.3. Evaluation Methods

TSR was visually assessed by two observers (BG, ZK) according to the recommendations of Mesker et al. [5] on digitized H&E-stained whole slide surgical sections. First, the most stroma-abundant area was selected after examining the entire tumor using low magnification. Afterward, an annotation was placed on the selected area with tumor cells present at all borders of the annotation. To represent the field of view of a conventional microscope using a 10× objective, a 3.14 mm^2^ circle area was selected. The stromal content of the annotated area was estimated in increments of 5%. According to the available literature, a cut-off value of 50% is considered discriminative for prognosis between stroma-low and stroma-high tumors [6].

To determine the OSR on the digitized slides, the entire tumor area was annotated, excluding areas of necrosis, mucus, and fibrotic foci. (Figure 6) The initial visual estimation of the OSR was conducted in increments of 5%.

The presence of fibrotic foci and necrosis were also noted on H&E slides when they exceeded 3.14 mm^2^.

**Figure 7:**
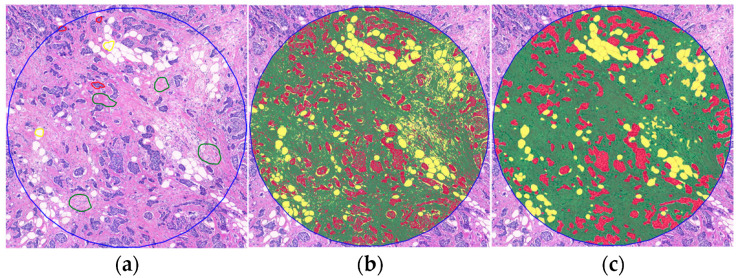
Steps to create a digital image analysis algorithm in PatternQuant using Slideviewer on an H&E-stained slide at 100× magnification: (**a**) annotating relevant training areas of tumor tissue (red), stroma (green), and cell-free area/background (yellow); (**b**) primary result; (**c**) result after contour smoothing.

DIA was conducted on the previously annotated areas for visual assessment in the PatternQuant submodule of Quantcenter (3DHISTECH) to evaluate TSR and OSR. Tumor tissues were divided into three clusters: tumor tissue (red), stroma (green), and cell-free area/background (yellow). The observers annotated the representative areas of each cluster. Following the algorithm training, measurements were conducted on the area of interest to determine the percentage of each cluster (Figure 7). TSR and OSR were calculated using the following formula: stroma/(stroma + tumor tissue) × 100, i.e., green/(green + red) × 100. The slides were analyzed twice by Observer 1 (ZK) and once by Observer 2 (BG), each time using different training areas. Due to the high intraobserver and interobserver correlation, the scores of the first evaluation were used.

The IHC reactions were evaluated using both visual assessment and DIA. Initially, the tumor tissue was annotated on the digitized TMA slides. Areas of necrosis, extensive mucus, and stroma of normal breast tissue were excluded from the analysis. The visual assessment involved estimating the proportion of positively stained fibrils in increments of 5%. Additionally, the staining intensity was categorized into three groups: weak, moderate, and strong.

**Figure 8:**
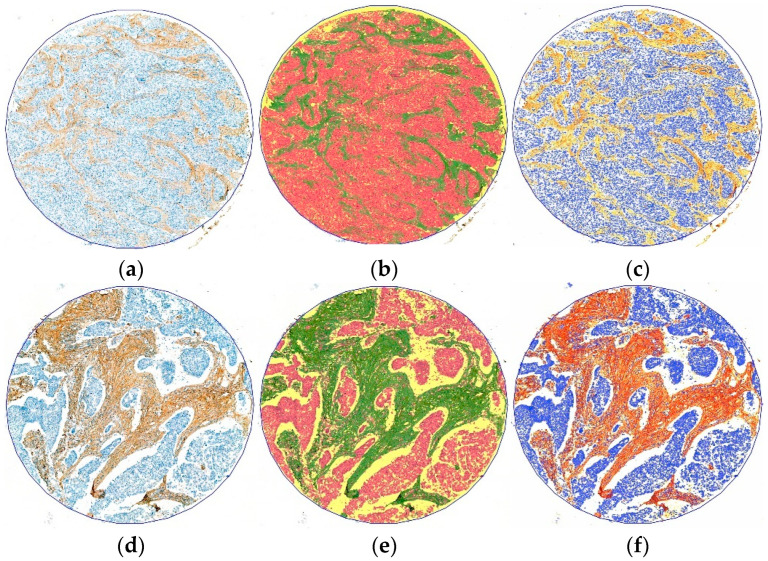
Comparative digital image analysis of type-III collagen immunohistochemical staining on TMA: PatternQuant and DensitoQuant assessments; (**a**,**d**) type-III collagen immunohistochemical stained TMA; (**b**,**e**) PatternQuant assessment of type-III collagen stained TMA; (**c**,**f**) DensitoQuant assessment of type-III collagen stained TMA.

As part of the digital analysis, the PatternQuant module of Quantcenter (3DHISTECH) was employed to measure the positively stained fibrils on TMAs with type-I collagen, type-III collagen, and fibrillin-1 IHC reactions (Figure 8). During the training process, three clusters were identified: tumor tissue (red), positively stained fibrils (green), and cell-free area/background (yellow). The same formula mentioned earlier was used to calculate the proportion of the brown reaction. Three to five annotated areas were used to define each cluster. To specify the cluster of positive IHC reactions, the observer annotated weakly, intermediately, and strongly stained areas. If the measurements from the visual and DIA assessments differed by more than 20%, the score of digital measurement was reevaluated.

Digital analysis of the type-III collagen and fibrillin-1 staining intensity was performed in the DensitoQuant module of Quantcenter (3DHISTECH) on the cluster of positively stained fibrils (Figure 8). The threshold was set on a brown scale with visual control for each immunoreaction. For type-III collagen, positivity was categorized as 0–6 (weak), 6–15 (moderate), and >15 (strong). For fibrillin-1, positivity was categorized as 0–8 (weak), 0–25 (moderate), and >25 (strong). Measurements were conducted on the area of interest to determine the extent of each intensity category. Based on the proportions of weak, moderate, and strong staining, the algorithm generated a score ranging from 100 to 300, with 100 representing entirely weakly positive stroma and 300 representing stroma consisting only of strong positive fibrils.

### 4.4. Comparison with Independent Cohorts

We compared our results with the datasets of Kaplan–Meier Plotter on both mRNA and quantitative proteome analysis of the genes of interest [42]. We selected the TNBC cases (ER status–IHC negative, PR status–IHC negative, HER2 status–array negative). For mRNA analysis, we used the JetSet best probe and auto-selected the best cut-off. For proteome analysis, we used the dataset of Liu_2014 with auto-select best cut-off.

### 4.5. Statistics

Statistical analyses were performed using IBM SPSS statistics (v29 for Windows). OS was defined as the time from the start of treatment to the date of death from any cause. If the patient was still alive at the end of the study period or lost to follow-up, OS was censored at the date of the last follow-up. Progression-free survival (PFS) was defined as the time from the start of treatment to the date of the first documented disease progression or recurrence, whichever occurred first. If the patient had not experienced disease progression, died at the end of the study period, or was lost to follow-up, PFS was censored at the date of the last follow-up.

To ensure the data followed a normal distribution, we first applied the Kolmogorov–Smirnov test. For the assessment of correlation between observers, we utilized both Pearson and Spearman correlation coefficients. Pearson correlation was selected to evaluate linear relationships for normally distributed data, while Spearman correlation was employed to account for potential non-linear associations in data that did not meet normality assumptions. Differences in clinicopathological characteristics between the stroma-high and stroma-low groups were analyzed using independent *t*-tests and chi-square (X^2^) tests. The independent *t*-test was applied to compare continuous variables, assuming normal distribution and equal variances between groups. In contrast, the chi-square test was chosen for categorical variables to assess the association between stroma status and clinical features.

Survival analyses were conducted using several methods to provide a comprehensive evaluation of patient outcomes. The Kaplan–Meier method, combined with the Log-Rank test, was employed to generate survival curves and compare survival distributions between groups. Additionally, univariate and multivariate Cox regression models were utilized to identify and adjust for potential confounders, providing hazard ratios and confidence intervals to quantify the impact of various factors on survival. To determine optimal cut-off points for the stroma markers, we initially used the median value. These cut-off points were then refined through multiple iterations of the Log-Rank test, allowing us to identify thresholds that most effectively distinguished between different prognostic groups.

## 5. Conclusions

In conclusion, in our TNBC cohort using digital assessment of H&E-stained whole slide images, we found that the stromal content of the whole tumor negatively correlates both with the OS (*p* = 0.044) and PFS (*p* = 0.052). Also, we identified promising extracellular matrix protein biomarkers that had not been previously examined in detail in TNBCs. Importantly, high levels of type-I collagen, type-III collagen, and fibrillin-1 extracellular matrix protein expression were found to be linked with worse OS (*p* = 0.004, *p* = 0.013, and *p* = 0.005, respectively) and PFS (*p* = 0.028, *p* = 0.025, and *p* = 0.002, respectively) in TNBC. Digital image analysis proved to be a valuable tool in reliably separating the tumor tissue from its adjacent microenvironment both in H&E-stained and particularly in matrix immunostained digital slides for the objective measurement of tumor–stroma and overall tumor–stroma ratios.

## Figures and Tables

**Table 1:** Clinicopathological features stratified by tumor–stroma ratio (TSR) # and overall stroma ratio (OSR) ## with *t*-test and Log-Rank test (p_LR_).

	All	Visual TSR			Visual OSR		
	*n* = 99	Stroma-High (>65)	Stroma-Low (≤65)	*t*-Test/Log-Rank (*p* *)	Stroma-High (≥35)	Stroma-Low (<35)	*t*-Test/Log-Rank (*p* *)
Age (*n* = 99)							
<48 years	17	5	12	t = 1.8 (0.07)	3	14	t = 3.5 (<0.001 **)
≥48 years	82	41	41		48	34	
pT category (*n* = 97)							
pT1b-pT1c	54	21	33	t = 0.83 (0.41)	27	27	t = 0.03 (0.97)
pT2-pT3	43	23	20		23	20	
pN category (*n* = 85)							
pN0-pN1mi	55	20	35	t = 3.3 (0.002 **)	25	30	t = 2.5 (0.013 *)
pN1-pN3a	30	19	11		20	10	
Stromal tumor-infiltrating lymphocyte (*n* = 99)							
≤20	50	27	23	t = 1.4 (0.15)	32	18	t = 3.0 (0.004 **)
>20	49	19	30		19	30	
Mitotic index (*n* = 85)							
<25	36	24	12	t = 3.0 (0.003 **)	25	11	t = 3.7 (0.001 **)
≥25	49	15	34		20	29	
Overall survival (*n* = 99)							
Alive	62	28	34	p_LR_ = 0.49	27	35	p_LR_ = 0.017 *
Dead	37	18	19		24	13	
Progression-free survival (*n* = 99)							
No progression	63	27	36	p_LR_ = 0.35	29	34	p_LR_ = 0.19
Progression	36	19	17		21	15	

* Correlation is significant at the 0.05 level. ** Correlation is significant at the 0.01 level. # Ratio of stroma and tumor on the most desmoplastic area of the tumor. ## Ratio of stroma and tumor on the entire tumor area.

**Table 2:** (A) Intraobserver and interobserver agreement on tumor–stroma ratio (TSR) using visual evaluation and digital image analysis (DIA), quantified by Spearman correlation. (B) Agreement of digital image analysis (DIA) and visual assessment on overall stromal ratio (OSR) results quantified by Pearson correlation.

**A**						
**Spearman Correlation**	**Visual TSR Obs. 1/1**	**Visual TSR Obs. 1/2**	**Visual TSR Obs. 2**	**DIA TSR Obs. 1/1**	**DIA TSR** **Obs. 1/2**	**DIA TSR** **Obs. 2**
Visual TSR Obs. 1/1	1	0.979 **	0.865 **	0.943 **	0.946 **	0.930 **
Visual TSR Obs. 1/2	0.979 **	1	0.866 **	0.928 **	0.943 **	0.911 **
Visual TSR Obs. 2	0.865 **	0.866 **	1	0.876 **	0.879 **	0.888 **
DIA TSR Obs. 1/1	0.943 **	0.928 **	0.876 **	1	0.961 **	0.964 **
DIA TSR Obs. 1/2	0.946 **	0.943 **	0.879 **	0.961 **	1	0.962 **
**B**						
**Pearson Correlation**			**Visual OSR**	**DIA OSR** **Obs. 1/1**	**DIA OSR** **Obs. 1/2**	**DIA OSR** **Obs. 2**
Visual OSR			1	0.833 **	0.833 **	0.843 **
DIA OSR Obs. 1/1			0.833 **	1	0.911 **	0.920 **
DIA OSR 2 Obs. 1/2			0.833 **	0.911 **	1	0.918 **

** Correlation is significant at the *p* = 0.01 level (2-tailed).

**Table 3:** Univariate and multivariate Cox regression results on overall stroma ratio (OSR), key clinicopathological parameters, and survival.

**PFS**	**Univariate HR**	**CI**	***p***	**Multivariate HR**	**CI**	***p***
DIA OSR (continuous)	1.012	0.996–1.034	0.113	1.007	0.984–1.031	0.540
DIA OSR categorical (≤39.34% vs. above)	1.907	0.979–3.715	0.058			
Number of positive lymph nodes	1.220	1.107–1.345	<0.001 **	1.179	1.065–1.306	0.002 **
Presence of fibrotic foci	2.119	1.007–4.458	0.048 *	2.043	0.894–4.670	0.090
sTIL categorical (≤20% vs. above)	0.498	0.252–0.985	0.045 *			
**OS**	**Univariate HR**	**CI**	***p***	**Multivariate HR**	**CI**	***p***
DIA OSR (continuous)	1.023	1.003–1.044	0.023 *	1.037	1.007–1.041	0.028 *
DIA OSR categorical (≤39.34% vs. above)	1.955	1.004–3.808	0.045 *			
Age	1.039	1.009–1.069	0.01 *	1.023	0.989–1.059	0.188
Number of positive lymph nodes	1.14	1.039–1.250	0.006 **	1.083	0.96–1.223	0.195
Presence of necrosis	2.739	1.043–7.191	0.041 *			
sTIL categorical (≤20 vs. above)	0.499	0.256–0.972	0.041 *	0.995	0.973–1.017	0.631
Mitotic index (MI)	1.017	0.097–1.038	0.096	1.040	1.040–1.015	0.002 **

Abbreviations: CI: confidence interval; DIA: digital image analysis; HR: hazard ratio; OSR: overall stroma ratio; OS: overall survival; PFS: progression-free survival; sTIL: stromal tumor-infiltrating lymphocytes. * Correlation is significant at the *p* = 0.05 level (2-tailed). ** Correlation is significant at the *p* = 0.01 level (2-tailed).

**Table 4:** Summary of the key characteristics derived from TMAs.

Characteristics of the TMAs (*n*)	H&E (86)	Type-I Collagen (84)	Type-III Collagen (83)	Fibrillin-1 (85)
Median percentage of positivity (range)	37.5 (5–80)	41.8 (5.3–85)	27.8 (1.7–85.4)	30 (2–71.6)
Cut-offs used for further calculations * (number or cases below; above)		OS, PFS: 45% (46; 35)	OS: 30% (37; 43) PFS: 45% (58; 22)	OS, PFS: 20% (32; 50)
Median intensity (range)			198 (126–270)	191 (143–261)
Median DQ * PQ (range)			5507 (309–18,444)	5050 (372–18,687)
Median type-I collagen rate (range)			0.76 (0.05–2.58)	0.64 (0.18–1.56)

* Best cut-off value determined utilizing multiple Log-Rank analysis. Abbreviations: DQ: DensitoQuant; H&E: hematoxylin and eosin-staining; PQ: PatternQuant; TMA: tissue microarray

**Table 5:** Correlation between the percentage of positivity for each immunostaining, evaluated with PatternQuant (PQ) on tissue microarrays (TMAs), and the overall stromal ratio (OSR) determined on whole slide images (WSI).

Pearson’s Correlation	Stromal Ratio (H&E TMA)	Type-I Collagen	Type-III Collagen	Fibrillin-1
Stromal ratio (H&E TMA)	1	0.864 **	0.718 **	0.727 **
Type-I collagen	0.864 **	1	0.811 **	0.809 **
Type-III collagen	0.718 **	0.811 **	1	0.793 **
Fibrillin-1	0.727 **	0.809 **	0.793 **	1
OSR (WSI)	0.551 **	0.627 **	0.603 **	0.590 **

** Correlation is significant at the *p* = 0.01 level (2-tailed).

**Table 6:** Significant Cox regression analysis results for collagen types I/III, fibrillin-1, and OS/PFS.

**OS**	**Univariate HR**	**CI**	***p***	**Multivariate HR**	**CI**	***p***
DIA type-I collagen categorical (≤45% vs. above)	2.826	1.340–5.961	0.006 **	3.075	1.309–7.228	0.01 *
DIA type-III collagen (continuous)	1.022	1.004–1.040	0.017 *	1.030	1.008–1.054	0.008 **
DIA type-III collagen categorical (≤30% vs. above)	2.697	1.269–5.729	0.01 *	3.467	1.411–8.522	0.007 **
DIA fibrillin-1 (continuous)	1.031	1.007–1.056	0.011 *	1.040	1.009–1.071	0.01 *
DIA fibrillin-1 categorical (≤20% vs. above)	3.129	1.331–7.354	0.009 **	3.439	1.248–9.479	0.017 *
**PFS**	**Univariate HR**	**CI**	***p***	**Multivariate HR**	**CI**	***p***
DIA type-I collagen categorical (≤45% vs. above)	2.255	1.068–4.760	0.033 *	2.725	1.197–2.725	0.017 *
DIA type-III collagen (continuous)	1.018	1.001–1.036	0.037 *	1.033	1.009–1.058	0.008 **
DIA type-III collagen categorical (≤30% vs. above)	2.280	1.087–4.785	0.029 *	3.825	1.364–10.731	0.011 *
DIA fibrillin-1 (continuous)	1.030	1.007–1.054	0.012 *	1.045	1.013–1.077	0.005 **
DIA fibrillin-1 categorical (≤20% vs. above)	3.388	1.372–8.368	0.008 **	3.815	1.275–11.574	0.018 *

Abbreviations: CI: confidence interval; DIA: digital image analysis; HR: hazard ratio; OS: overall survival; PFS: progression-free survival. * Correlation is significant at the *p* = 0.05 level (2-tailed). ** Correlation is significant at the *p* = 0.01 level (2-tailed).

## Data Availability

Data supporting reported results are avalaible on reasonable request from the corresponding author.

## Supplementary Materials

The following supporting information can be downloaded at https://www.mdpi.com/article/10.3390/ijms25179445/s1.

## References

[1] Lukasiewicz S., Czeczelewski M., Forma A., Baj J., Sitarz R., Stanislawek A. (2021). Breast Cancer-Epidemiology, Risk Factors, Classification, Prognostic Markers, and Current Treatment Strategies-An Updated Review. Cancers.

[2] Ferlay J., Ervik M., Lam F., Colombet M., Mery L., Piñeros M., Znaor A., Soerjomataram I., Bray F. (2018). Global Cancer Observatory: Cancer Today.

[3] Goldhirsch A., Winer E.P., Coates A.S., Gelber R.D., Piccart-Gebhart M., Thurlimann B., Senn H.J. (2013). Personalizing the treatment of women with early breast cancer: Highlights of the St Gallen International Expert Consensus on the Primary Therapy of Early Breast Cancer 2013. Ann. Oncol..

[4] Mehanna J., Haddad F.G., Eid R., Lambertini M., Kourie H.R. (2019). Triple-negative breast cancer: Current perspective on the evolving therapeutic landscape. Int. J. Womens Health.

[5] Mesker W.E., Junggeburt J.M., Szuhai K., de Heer P., Morreau H., Tanke H.J., Tollenaar R.A. (2007). The carcinoma-stromal ratio of colon carcinoma is an independent factor for survival compared to lymph node status and tumor stage. Cell. Oncol..

[6] Hagenaars S.C., Vangangelt K.M.H., Van Pelt G.W., Karancsi Z., Tollenaar R., Green A.R., Rakha E.A., Kulka J., Mesker W.E. (2022). Standardization of the tumor-stroma ratio scoring method for breast cancer research. Breast Cancer Res. Treat..

[7] Zhang X.L., Jiang C., Zhang Z.X., Liu F., Zhang F., Cheng Y.F. (2014). The tumor-stroma ratio is an independent predictor for survival in nasopharyngeal cancer. Oncol. Res. Treat..

[8] Wang K., Ma W., Wang J., Yu L., Zhang X., Wang Z., Tan B., Wang N., Bai B., Yang S. (2012). Tumor-stroma ratio is an independent predictor for survival in esophageal squamous cell carcinoma. J. Thorac. Oncol..

[9] Liu J., Liu J., Li J., Chen Y., Guan X., Wu X., Hao C., Sun Y., Wang Y., Wang X. (2014). Tumor-stroma ratio is an independent predictor for survival in early cervical carcinoma. Gynecol. Oncol..

[10] Chen Y., Zhang L., Liu W., Liu X. (2015). Prognostic Significance of the Tumor-Stroma Ratio in Epithelial Ovarian Cancer. BioMed Res. Int..

[11] Lv Z., Cai X., Weng X., Xiao H., Du C., Cheng J., Zhou L., Xie H., Sun K., Wu J. (2015). Tumor-stroma ratio is a prognostic factor for survival in hepatocellular carcinoma patients after liver resection or transplantation. Surgery.

[12] Wu J., Liang C., Chen M., Su W. (2016). Association between tumor-stroma ratio and prognosis in solid tumor patients: A systematic review and meta-analysis. Oncotarget.

[13] Karancsi Z., Hagenaars S.C., Nemeth K., Mesker W.E., Tokes A.M., Kulka J. (2023). Tumour-stroma ratio (TSR) in breast cancer: Comparison of scoring core biopsies versus resection specimens. Virchows Arch..

[14] Kramer C.J.H., Vangangelt K.M.H., van Pelt G.W., Dekker T.J.A., Tollenaar R., Mesker W.E. (2019). The prognostic value of tumour-stroma ratio in primary breast cancer with special attention to triple-negative tumours: A review. Breast Cancer Res. Treat..

[15] Vangangelt K.M.H., Green A.R., Heemskerk I.M.F., Cohen D., van Pelt G.W., Sobral-Leite M., Schmidt M.K., Putter H., Rakha E.A., Tollenaar R. (2020). The prognostic value of the tumor-stroma ratio is most discriminative in patients with grade III or triple-negative breast cancer. Int. J. Cancer.

[16] Moorman A.M., Vink R., Heijmans H.J., van der Palen J., Kouwenhoven E.A. (2012). The prognostic value of tumour-stroma ratio in triple-negative breast cancer. Eur. J. Surg. Oncol..

[17] Fisher T.B., Saini G., Rekha T.S., Krishnamurthy J., Bhattarai S., Callagy G., Webber M., Janssen E.A.M., Kong J., Aneja R. (2024). Digital image analysis and machine learning-assisted prediction of neoadjuvant chemotherapy response in triple-negative breast cancer. Breast Cancer Res..

[18] Jakab A., Patai A.V., Micsik T. (2022). Digital image analysis provides robust tissue microenvironment-based prognosticators in patients with stage I-IV colorectal cancer. Hum. Pathol..

[19] Millar E.K., Browne L.H., Beretov J., Lee K., Lynch J., Swarbrick A., Graham P.H. (2020). Tumour Stroma Ratio Assessment Using Digital Image Analysis Predicts Survival in Triple Negative and Luminal Breast Cancer. Cancers.

[20] Atallah N.M., Wahab N., Toss M.S., Makhlouf S., Ibrahim A.Y., Lashen A.G., Ghannam S., Mongan N.P., Jahanifar M., Graham S. (2023). Deciphering the Morphology of Tumor-Stromal Features in Invasive Breast Cancer Using Artificial Intelligence. Mod. Pathol..

[21] Micke P., Strell C., Mattsson J., Martin-Bernabe A., Brunnstrom H., Huvila J., Sund M., Warnberg F., Ponten F., Glimelius B. (2021). The prognostic impact of the tumour stroma fraction: A machine learning-based analysis in 16 human solid tumour types. eBioMedicine.

[22] Yan D., Ju X., Luo B., Guan F., He H., Yan H., Yuan J. (2022). Tumour stroma ratio is a potential predictor for 5-year disease-free survival in breast cancer. BMC Cancer.

[23] Albusayli R., Graham J.D., Pathmanathan N., Shaban M., Raza S.E.A., Minhas F., Armes J.E., Rajpoot N. (2023). Artificial intelligence-based digital scores of stromal tumour-infiltrating lymphocytes and tumour-associated stroma predict disease-specific survival in triple-negative breast cancer. J. Pathol..

[24] de Visser K.E., Joyce J.A. (2023). The evolving tumor microenvironment: From cancer initiation to metastatic outgrowth. Cancer Cell.

[25] Oskarsson T. (2013). Extracellular matrix components in breast cancer progression and metastasis. Breast.

[26] Halper J. (2021). Basic Components of Connective Tissues and Extracellular Matrix: Fibronectin, Fibrinogen, Laminin, Elastin, Fibrillins, Fibulins, Matrilins, Tenascins and Thrombospondins. Adv. Exp. Med. Biol..

[27] Popova N.V., Jucker M. (2022). The Functional Role of Extracellular Matrix Proteins in Cancer. Cancers.

[28] Furler R.L., Nixon D.F., Brantner C.A., Popratiloff A., Uittenbogaart C.H. (2018). TGF-beta Sustains Tumor Progression through Biochemical and Mechanical Signal Transduction. Cancers.

[29] Meng C., He Y., Wei Z., Lu Y., Du F., Ou G., Wang N., Luo X.G., Ma W., Zhang T.C. (2018). MRTF-A mediates the activation of COL1A1 expression stimulated by multiple signaling pathways in human breast cancer cells. Biomed. Pharmacother..

[30] Hsu K.S., Dunleavey J.M., Szot C., Yang L., Hilton M.B., Morris K., Seaman S., Feng Y., Lutz E.M., Koogle R. (2022). Cancer cell survival depends on collagen uptake into tumor-associated stroma. Nat. Commun..

[31] Liu J., Shen J.X., Wu H.T., Li X.L., Wen X.F., Du C.W., Zhang G.J. (2018). Collagen 1A1 (COL1A1) promotes metastasis of breast cancer and is a potential therapeutic target. Discov. Med..

[32] Kauppila S., Stenback F., Risteli J., Jukkola A., Risteli L. (1998). Aberrant type I and type III collagen gene expression in human breast cancer in vivo. J. Pathol..

[33] Kim S.H., Lee H.Y., Jung S.P., Kim S., Lee J.E., Nam S.J., Bae J.W. (2014). Role of secreted type I collagen derived from stromal cells in two breast cancer cell lines. Oncol. Lett..

[34] Singh D., Rai V., Agrawal D.K. (2023). Regulation of Collagen I and Collagen III in Tissue Injury and Regeneration. Cardiol. Cardiovasc. Med..

[35] Brisson B.K., Dekky B., Berger A.C., Mauldin E.A., Loebel C., Yen W., Stewart D.C., Gillette D., Assenmacher C.A., Cukierman E. (2023). Tumor-restrictive type III collagen in the breast cancer microenvironment: Prognostic and therapeutic implications. Res. Sq..

[36] Brisson B.K., Mauldin E.A., Lei W., Vogel L.K., Power A.M., Lo A., Dopkin D., Khanna C., Wells R.G., Pure E. (2015). Type III Collagen Directs Stromal Organization and Limits Metastasis in a Murine Model of Breast Cancer. Am. J. Pathol..

[37] Zhang L., Wang L., Yang H., Li C., Fang C. (2021). Identification of potential genes related to breast cancer brain metastasis in breast cancer patients. Biosci. Rep..

[38] Yang F., Lin L., Li X., Wen R., Zhang X. (2022). Silencing of COL3A1 represses proliferation, migration, invasion, and immune escape of triple negative breast cancer cells via down-regulating PD-L1 expression. Cell Biol. Int..

[39] Thomson J., Singh M., Eckersley A., Cain S.A., Sherratt M.J., Baldock C. (2019). Fibrillin microfibrils and elastic fibre proteins: Functional interactions and extracellular regulation of growth factors. Semin. Cell Dev. Biol..

[40] Wang Z., Liu Y., Lu L., Yang L., Yin S., Wang Y., Qi Z., Meng J., Zang R., Yang G. (2015). Fibrillin-1, induced by Aurora-A but inhibited by BRCA2, promotes ovarian cancer metastasis. Oncotarget.

[41] Brierley J.D., Gospodarowicz M.K., Wittekind C. (2016). TNM Classification of Malignant Tumours.

[42] Gyorffy B. (2021). Survival analysis across the entire transcriptome identifies biomarkers with the highest prognostic power in breast cancer. Comput. Struct. Biotechnol. J..

[43] de Kruijf E.M., van Nes J.G., van de Velde C.J., Putter H., Smit V.T., Liefers G.J., Kuppen P.J., Tollenaar R.A., Mesker W.E. (2011). Tumor-stroma ratio in the primary tumor is a prognostic factor in early breast cancer patients, especially in triple-negative carcinoma patients. Breast Cancer Res. Treat..

[44] Xu Z., Li Y., Wang Y., Zhang S., Huang Y., Yao S., Han C., Pan X., Shi Z., Mao Y. (2021). A deep learning quantified stroma-immune score to predict survival of patients with stage II-III colorectal cancer. Cancer Cell Int..

[45] Zhao K., Li Z., Yao S., Wang Y., Wu X., Xu Z., Wu L., Huang Y., Liang C., Liu Z. (2020). Artificial intelligence quantified tumour-stroma ratio is an independent predictor for overall survival in resectable colorectal cancer. eBioMedicine.

[46] Acerbi I., Cassereau L., Dean I., Shi Q., Au A., Park C., Chen Y.Y., Liphardt J., Hwang E.S., Weaver V.M. (2015). Human breast cancer invasion and aggression correlates with ECM stiffening and immune cell infiltration. Integr. Biol..

[47] Kaushik S., Pickup M.W., Weaver V.M. (2016). From transformation to metastasis: Deconstructing the extracellular matrix in breast cancer. Cancer Metastasis Rev..

[48] Li X., Jin Y., Xue J. (2024). Unveiling Collagen’s Role in Breast Cancer: Insights into Expression Patterns, Functions and Clinical Implications. Int. J. Gen. Med..

[49] Jansson M., Lindberg J., Rask G., Svensson J., Billing O., Nazemroaya A., Berglund A., Warnberg F., Sund M. (2024). Stromal Type I Collagen in Breast Cancer: Correlation to Prognostic Biomarkers and Prediction of Chemotherapy Response. Clin. Breast Cancer.

[50] Huang J., Zhang L., Wan D., Zhou L., Zheng S., Lin S., Qiao Y. (2021). Extracellular matrix and its therapeutic potential for cancer treatment. Signal Transduct. Target. Ther..

[51] Li L., Huang J., Liu Y. (2023). The extracellular matrix glycoprotein fibrillin-1 in health and disease. Front. Cell Dev. Biol..

[52] Bartha A., Gyorffy B. (2021). TNMplot.com: A Web Tool for the Comparison of Gene Expression in Normal, Tumor and Metastatic Tissues. Int. J. Mol. Sci..

[53] Cizkova K., Foltynkova T., Gachechiladze M., Tauber Z. (2021). Comparative Analysis of Immunohistochemical Staining Intensity Determined by Light Microscopy, ImageJ and QuPath in Placental Hofbauer Cells. Acta Histochem. Cytochem..

[54] Szekely T., Wichmann B., Maros M.E., Csizmadia A., Bodor C., Timar B., Krenacs T. (2023). Myelofibrosis progression grading based on type I and type III collagen and fibrillin 1 expression boosted by whole slide image analysis. Histopathology.

