# Supplementary material for: Digital Whole Slide Image Analysis of Elevated Stromal Content and Extracellular Matrix Protein Expression Predicts Adverse Prognosis in Triple-Negative Breast Cancer
Source: Int J Mol Sci. 2024 Aug 30;25(17):9445. doi: 10.3390/ijms25179445 (PMC11394775; doi:10.3390/ijms25179445)
Supplement: Supplementary file 1 [file ijms-25-09445-s001.zip › Supplementary figure 1.pdf]

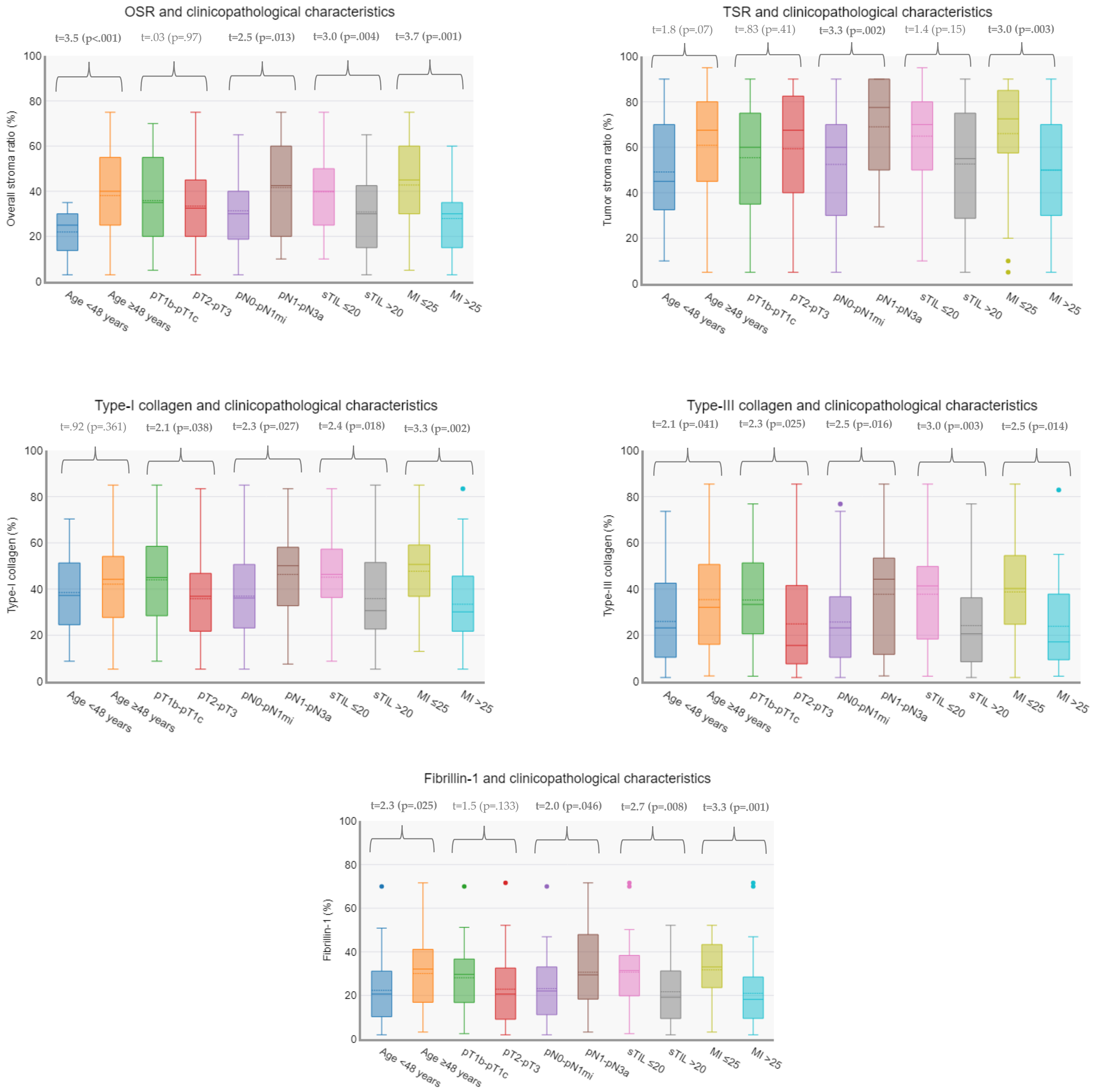

Figure S1. Boxplot figure visualizing the correlation between the percentage values of the evaluated stromal variables (TSR, OSR, type-I collagen, type-III collagen, fibrillin-1) and the most important clinicopathological characteristics (https://www.statskingdom.com/advanced-boxplot-maker.html). The dashed line represents the mean, and the solid line represents the median value. P-values below .05 are in bold.

Abbreviations: MI: mitotic index, OSR: overall stroma ratio, sTIL: stromal tumor-infiltrating lymphocytes, TSR: tumor-stroma ratio
