# Supplementary material for: Digital Whole Slide Image Analysis of Elevated Stromal Content and Extracellular Matrix Protein Expression Predicts Adverse Prognosis in Triple-Negative Breast Cancer
Source: Int J Mol Sci. 2024 Aug 30;25(17):9445. doi: 10.3390/ijms25179445 (PMC11394775; doi:10.3390/ijms25179445)
Supplement: Supplementary file 1 [file ijms-25-09445-s001.zip › Supplementary table 1.pdf]

Table S1. Correlation between the percentage of positivity values of each immunostaining evaluated visually and with PatternQuant (PQ) on TMAs

**Pearson correlation**	Type-I collagen PQ	Type-III collagen PQ	Fibrillin-1 PQ
Type-I collagen visual (%)	.951**		
Type-III collagen visual (%)		.969**	
Fibrillin-1 visual (%)			.927**

**Correlation is significant at the p=.01 level (2-tailed)
