# Supplementary material for: Digital Whole Slide Image Analysis of Elevated Stromal Content and Extracellular Matrix Protein Expression Predicts Adverse Prognosis in Triple-Negative Breast Cancer
Source: Int J Mol Sci. 2024 Aug 30;25(17):9445. doi: 10.3390/ijms25179445 (PMC11394775; doi:10.3390/ijms25179445)
Supplement: Supplementary file 1 [file ijms-25-09445-s001.zip › Supplementary table 2.pdf]

Table S2. Correlation between the intensity values of each immunostaining evaluated visually and with DensitoQuant (DQ) on TMAs

**Cohen's Kappa**	Type-III collagen DQ	Fibrillin-1 DQ
Type-III collagen visual (intensity)	.566**	
Fibrillin-1 visual (intensity)		.398**

**Correlation is significant at the p=.01 level (2-tailed)
